# Studying Brown Adipose Tissue in a Human *in vitro* Context

**DOI:** 10.3389/fendo.2020.00629

**Published:** 2020-09-15

**Authors:** Isabella Samuelson, Antonio Vidal-Puig

**Affiliations:** ^1^Metabolic Research Laboratories, University of Cambridge, Cambridge, United Kingdom; ^2^Department of Cellular Genetics, Wellcome Sanger Institute (WT), Hinxton, United Kingdom

**Keywords:** brown adipose tissue (BAT), 3D culture, HPSC model, obesity, thermogenic adipocyte differentiation, brown adipogenesis, human adipocytes, beige adipocyte

## Abstract

New treatments for obesity and associated metabolic disease are increasingly warranted with the growth of the obesity pandemic. Brown adipose tissue (BAT) may represent a promising therapeutic target to treat obesity, as this tissue has been shown to regulate energy expenditure through non-shivering thermogenesis. Three different strategies could be employed for therapeutic targeting of human thermogenic adipocytes: increasing BAT mass through stimulation of BAT progenitors, increasing BAT function through regulatory pathways, and increasing WAT browning through promotion of beige adipocyte formation. However, these strategies require deeper understanding of human brown and beige adipocytes. While murine studies have greatly increased our understanding of BAT, it is becoming clear that human BAT does not exactly resemble that of the mouse, highlighting the need for human *in vitro* models of brown adipocytes. Several different human brown adipocyte models will be discussed here, along with the potential to improve brown adipocyte culture through recreation of the BAT microenvironment.

## The Obesity Pandemic

Obesity, defined as excessive fat accumulation, has reached pandemic proportions. Among adults, more than 650 million were obese in 2016 (BMI ≥ 30), with over 340 million obese or overweight (BMI ≥ 25) children and adolescents (1). Obesity is associated with the metabolic syndrome, which comprises a cluster of metabolic abnormalities including insulin resistance, obesity, dyslipidemia, and hypertension, which are risk factors for cardiovascular disease and diabetes (2). Furthermore, obesity is a risk factor for cancer and musculoskeletal disorders, including osteoarthritis, among other diseases (3, 4). Thus, obesity places a substantial burden on society and healthcare systems and despite extensive research into the mechanisms controlling energy balance, body weight, and appetite, safe and effective measures to treat and prevent obesity are lacking.

## Brown Adipose Tissue

Brown adipose tissue (BAT) has become a focus area for weight loss therapies. This tissue, evolved to help maintain core body temperature, is able to burn nutrients as heat, thereby increasing total energy expenditure (5). This process is known as non-shivering thermogenesis and is mediated by the mitochondrial uncoupling protein1 (UCP1), and BAT activation leads to increased energy expenditure and decreased metabolic efficiency.

BAT has been extensively studied in the mouse, and these studies have revealed an important role for BAT in the control of energy balance and whole-body metabolism. BAT activation has, for instance, been shown to positively influence plasma triglyceride and cholesterol levels, atherosclerosis development, glucose tolerance, insulin sensitivity, and hepatic steatosis, in addition to promoting weight loss (6–9).

## Beige Adipose Tissue

Another type of adipose tissue (AT), which has received considerable attention for the treatment of obesity, is beige AT, which arises from white adipose tissue (WAT) browning. WAT browning is characterized by the presence of multilocular, brown adipocyte (BA)-marker expressing adipocytes within WAT—so-called beige or brite adipocytes. Beige adipocytes can appear in WAT, mainly subcutaneous (sc)WAT, after cold exposure, hypercaloric diet, β-adrenergic stimulation, and exercise (10), and are believed to influence metabolism similarly to BAT (11, 12). Although beige adipocytes show high expression of UCP1 (13), they are believed to originate from different lineages than BAs (14), although this is still debated.

## BAT in Humans—Beige or Brown?

In humans, BAT mass and prevalence have been found to correlate negatively with BMI, diabetes status, and glucose plasma levels (15), and cold-induced BAT activation also had beneficial metabolic effects and induced weight loss (16, 17), suggesting that human and murine BAT may have similar metabolic roles. It has been estimated that adult lean men may have around 330 ml of cold-inducible BAT, with lower volumes (around 130 ml) found in obese men (18). Furthermore, seasonal changes in WAT thermogenic gene expression have been described (19), suggesting that human WAT has the capacity for browning, although the impact of browning on whole-body metabolism is unclear.

Based on this ability of BAT to increase energy expenditure and improve metabolic function, BAT seems a likely target for the development of obesity therapeutics. Importantly, the efficacy of these therapeutics depends on whether activation of BAT in adults is able to significantly influence energy expenditure. Cold exposure in adults is able to increase energy expenditure varying from 20 kcal/day (20) to more than 100 kcal/day (17), and this can be increased further through chronic cold adaptation or increased BAT mass (17, 18).

A growing body of research is focusing on identifying genes and molecules able to influence BAT biology; however, one question is how well the findings in mice translate to humans. First, there is debate around whether human BAT resembles murine brown or beige AT more closely. Analyses of human neck fat have revealed that on a molecular level, deep neck fat mostly resembles that of murine canonical BAT, whereas the superficial neck fat may be more white or beige (21). In a study characterizing beige adipocytes, Wu et al. (13) generated murine brown and beige adipocyte gene expression signatures. Comparing these signatures with human supraclavicular BAT, they found that human BAT is closer to murine beige than brown AT. A similar study comparing murine AT gene signatures with human BAT from multiple anatomical locations came to the same conclusion (22). In contrast, a recent study suggests that when mice are physiologically humanized, meaning housed at thermoneutrality, fed a “Western diet,” and examined at 9–11 months old, their BAT becomes comparable with human supraclavicular BAT (23), although this conclusion has raised some discussion (24). This debate may likely be solved as the composition of human and murine brown and beige AT is better characterized with the help of more sophisticated techniques such as single-cell RNA sequencing. Owing to discrepancies between mRNA and protein expression levels of certain genes, including *UCP1* (25), purely transcriptomic analysis of different BA populations may not be adequate to fully characterize their identity and thermogenic potential. To this end, protein analysis and functional characterization, including β-adrenergic response and bioenergetics, are required.

Second, whereas treatment with β_3_-adrenergic receptor (β_3_AR) agonists in mice is able to selectively stimulate BAT and protect against diet-induced obesity, these effects are not seen in humans at doses low enough to prevent cardiovascular side effects (26, 27). Interestingly, a recent study found that the β_1_AR may be the predominant βAR in human BAT (28), highlighting another confounding issue when translating murine BAT studies to human biology. Other attempts to selectively activate thermogenesis through sympathomimetic drugs or mitochondrial uncouplers have not been successful (26). Thus, to achieve selective and safe activation of BAT in humans, we may need to look beyond adrenergic stimulation or uncoupling mechanisms and instead focus on alternative pathways to target BAT biology. Specifically, rather than focusing exclusively on increasing the activation of existing BAT, other strategies could include increasing BAT mass through differentiation of progenitors, or increasing thermogenic activity in WAT through WAT browning.

## Strategies for the Targeting of Human BAT

Increased energy expenditure in humans through BAT thermogenesis could be achieved by (a) increasing functional BAT mass, (b) increasing BAT activity, and (c) increasing WAT browning.

### Increased BAT Mass/Recruitment

Increased BAT mass could be achieved through the recruitment of BA progenitors. As mentioned earlier, decreased BAT mass is found in obese individuals—the very individuals the BAT therapeutics are intended for—and thus even if a method to selectively activate BAT was found, this may be of limited use to obese recipients. However, BAT activity can be enhanced through cold acclimation even in obese individuals (29), suggesting the presence of BA precursors, the differentiation of which may be promoted given appropriate stimulation. The development of targeted approaches to induce differentiation of BA progenitors requires in-depth knowledge of the developmental mechanisms of human BAT, which currently does not exist. Furthermore, newly differentiated BAs would still require activation through sympathetic or alternative pathways.

### Increased BAT Activity

Increased BAT activity, as discussed earlier, has been challenging to achieve without cardiovascular side effects. However, several molecules have been identified as possible therapeutic targets for the activation of BAT, reviewed in (30). First, members of the bone morphogenetic protein (BMP) family have been reported to play roles in BAT or WAT browning, mainly using murine models. These include, among others, BMP4, which may promote browning of WAT (31, 32), BMP7, which can promote BAT development and activation (33) as well as white adipocyte (WA) browning (31), and BMP8B, which was found to increase the adrenergic response of BAT and induce scWAT browning (34, 35). In addition, factors released from heart, muscle, liver, and immune cells have been shown to regulate thermogenesis (30). Although these studies reveal several candidate targets, most of them have been conducted in murine models or *in vitro*, and tangible therapeutics are still far from a reality.

### Increased WAT Browning

Finally, increased WAT browning is a strategy which may have substantial potential given the high abundance of WAT compared with BAT. However, as for BAs, the safe activation of beige adipocytes likely requires identification of specific molecular pathways to induce WAT browning that do not rely directly on adrenergic or uncoupling actions. These include fibroblast growth factor 21 (FGF21), which may regulate browning of scWAT through activation of peroxisome proliferator-activated receptor gamma coactivator 1-alpha (PGC1α) (36), as well as through UCP1-independent mechanisms (37). Another potential molecule is irisin, which may activate UCP1 in scWAT via p38-MAPK/ERK pathways (38), although the levels of circulating irisin and its metabolic effects in humans are debated (39, 40). In addition, more studies on human WAT browning are necessary to determine whether this process is able to influence whole-body energy expenditure in a notable manner.

The three strategies presented earlier are all limited by the lack of knowledge regarding human thermogenic adipocyte development and function. This lack of knowledge stems principally from the lack of robust *in vitro* models of human BAs.

## *In vitro* Models of Human BAT

**Figure 1:**
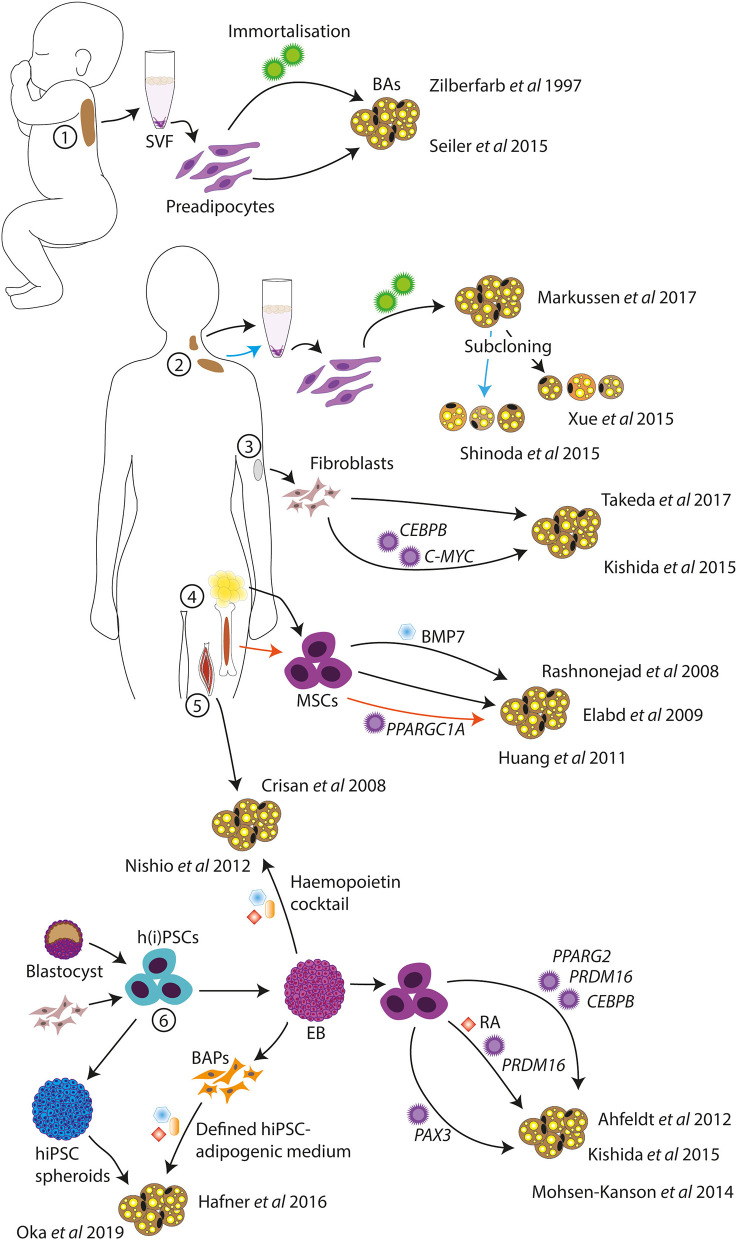
Schematic of *in vitro* human BA models. Human BA models have been generated from infant BAT (1), deep neck and supraclavicular BAT (2), dermal fibroblasts (3), MSCs from AT and bone marrow (4), skeletal muscle progenitor cells (5), and hPSCs (6). Standard adipogenic cocktails or variations were used for the differentiations unless otherwise indicated. SVF, stromal vascular fraction; BAs, brown adipocytes; MSCs, mesenchymal stem cells; h(i)PSCs, human (induced) pluripotent stem cells; BAPs, BA progenitors; EB, embryoid body; RA, retinoic acid.

Models of human BAs have been developed by several different groups and from starting material such as primary BAT, fibroblasts, muscle cells, adult stem cells, and pluripotent stem cells, among others. Some of these models are discussed later and represented in Figure 1.

### Primary Cell-Derived BA Models

The study of human primary BAT is limited by the scarceness of the disperse tissue and the need for invasive procedures to obtain biopsies. Nonetheless, several *in vitro* BA models derived from primary human BAT do exist. If one does have access to primary BAT, preadipocytes from non-viable human fetal interscapular BAT can be cultured and differentiated *in vitro* (41); however, these are not stable cell lines and are thus not a viable long-term solution for the study of human BAT. To this end, immortalized human BA lines may be of more use. One of these is the PAZ6 cell line from immortalized infant BAT vascular stromal cells (42). These cells readily differentiate to BAs and respond to β-adrenergic stimulation; however, the differentiation potential of these cells declines with increasing passage number, significantly limiting their experimental value. Similarly, subcloned immortalized BA-like cell lines have been generated from adult supraclavicular AT stromal vascular fraction (SVF) (43) and neck AT SVF (44). In addition, a more heterogeneous BA-like cell model was created from adult deep neck AT SVF without subcloning (45). These BA cell models demonstrated expression of *UCP1*, lipolytic response to cAMP (43), and increased oxygen consumption in response to β-adrenergic stimulation (44, 45), and two of the lines were maintained for at least 20 passages (44, 45). These cell lines thus represent useful models for the study of human BAT. However, their generation does require access to primary tissue, and they, therefore, come from limited starting material, which means that these cell lines will deplete eventually.

Rather than using primary BAT, BA-like cells can be directly generated from human dermal fibroblasts, eliminating the need for invasive biopsies. One study demonstrated that overexpression of CCAAT/enhancer binding protein beta (*CEBPB*) and avian myelocytomatosis viral oncogene homolog (*C-MYC*), coupled with an adipogenic cocktail, induced conversion of human fibroblasts to BA-like cells (46). Another study demonstrated the direct conversion of primary fibroblasts into BA-like cells with only chemical compounds, without using gene transfer (47). These compounds included rosiglitazone, which is an agonist of peroxisome proliferator-activated receptor gamma (PPARγ), and known to promote browning (48).

Alternatively, BA-like cells were shown to be differentiated from fetal and adult muscle progenitor cells expressing CD34, a marker of hematopoietic progenitors as well as vascular endothelial progenitors (49), thus supporting the common lineage of skeletal muscle and BAT. This technique does require invasive biopsies; however, muscle tissue may be more accessible than BAT.

In addition to primary tissue access, a limitation of the aforementioned models is the inherent risk of cell depletion. Thus, to circumvent this limitation, cell lines can be generated from human stem cells.

### Generation of Adipocytes From Stem Cells

#### Multipotent Stem Cell–Derived BAs

Multipotent mesenchymal stem cells (MSCs) are adult stem cells able to differentiate into a range of mesodermal cell types including adipocytes, and can be isolated from several tissues such as bone marrow, blood, and AT (50). MSCs isolated from human AT (termed human multipotent adipose-derived stem (hMADS) cells) could be differentiated to BA-like cells when stimulated with a PPARγ agonist on top of an adipogenic cocktail (51), and with the addition of BMP7 (52). Similarly, bone marrow–derived MSCs may also be able to differentiate to BA-like cells when overexpressing *PPARGC1A* (53). Thus, MSCs are a useful tool for the generation of BA-like cells, and the self-renewal ability of MSCs gives unlimited starting material, at least theoretically. As an alternative to MSCs, human pluripotent stem cells (hPSCs) have been used for the generation of BA cell models.

#### Pluripotent Stem Cell–Derived BAs

hPSCs are advantageous for the generation of cell models for several reasons, including that they are relatively easy to genetically engineer. Because of their pluripotency and the fact that protocols exist to differentiate hPSCs into a wide range of cell types (54), it is possible to generate isogenic disease models of cells from all three embryonic germ layers. Furthermore, the use of human induced (hi)PSCs, which are reprogrammed from human somatic cells (55), enables the generation of disease models with patient-specific genetic backgrounds.

Some studies have demonstrated the generation of adipocytes from hPSCs. Mature white and brown adipocytes were generated from hPSCs through transfection with lentiviral constructs to overexpress *PPARG2* or *PPARG2, CEBPB*, and *PRDM16*, respectively (56). In this study, hPSCs were first differentiated into MSCs using embryoid bodies (EBs), after which transgene expression was induced and an adipogenic cocktail was given. A similar method was used to generate BAs from hiPSCs through transduction of *PRDM16* (46). Here, EBs were generated in the presence of retinoic acid (RA), to obtain more myoblast-like cells (57), accounting for the reduced transgene requirement. Overexpression of transgenes in hPSCs is an efficient method for the generation and study of mature cell types (58–60), although it can be argued that this method of differentiation may not follow natural developmental steps and may instead shunt some of these pathways. Thus, this technique may not be optimal for the generation of a human BA model for the study of BA development.

Another group demonstrated the differentiation of BA progenitors from hiPSCs through EB formation and adipogenic cocktails (61). The differentiation efficiency was reported to be low but could be improved through overexpression of paired box 3 (*PAX3*), a marker of BA progenitors (61). A similar method was used to generate BA progenitors from hiPSCs without gene transfer, although this study reports higher differentiation yield, possibly due to inhibition of TGFβ signaling (62). TGFβ signaling has been shown to induce the conversion of endothelial cells to a more mesenchymal phenotype (63), and these findings, therefore, suggest an important role of endothelial cells in the adipogenic niche. The generation of BA-like cells from hPSCs using EB formation and a hemopoietin cocktail has also been reported (64), which may suggest that the developmental origin of BAs is more diverse than previously believed. Finally, BA-like cells were generated from hiPSCs in cytokine-free medium through the formation of spheroids (65), although the molecular mechanisms of this differentiation method remain unclear.

Although hPSCs can, in theory, give rise to any cell type of the human body, their immature/embryonic state means that it can be difficult to generate mature cell types from hPSCs without forward programming. This issue does not arise when using BA models derived from primary adult AT. As an alternative, hPSC-derived BAs can be matured *in vivo* by transplantation into mice, which is also a method of functional validation of the generated BAs (46, 64, 65). Currently, transplantation *in vivo* may be the best technique for recapitulation of the physiological BAT microenvironment; however, advances are also being made toward recreating BAT *in vitro* using 3D cell cultures.

## Recapitulating the BA Microenvironment *in vitro*

Long-term culture of adipocytes *in vitro* can be difficult because of the buoyancy of mature, lipid-laden adipocytes, which makes their attachment to 2D cell culture plates difficult (66). Furthermore, true understanding of BAT biology requires recapitulation of the *in vivo* BA microenvironment—the BA niche. *In vivo*, BAT is a highly vascularized and innervated tissue, containing not only BAs but also endothelial cells, fibroblasts, progenitor cells, and immune cells (5, 30). Furthermore, the extracellular matrix (ECM) of the AT is vital as a structural scaffold and to facilitate adipocyte differentiation, AT expansion, mechanotransduction, and biomolecule signaling (67–69).

### Culturing BAs in 3D

A first step toward recapitulating BAT *in vitro* may be to use 3D cultures, which can provide mechanical support, allow physiological cell organization, and prevent loss of mature adipocytes (70). 3D culture techniques are successful for WA culture (71), allowing *in vitro* culture of unilocular primary mature human WAs (72, 73). Unilocular WAs can also develop *in vitro* when differentiated in spheroids (74–76).

Fewer 3D culture systems have been tested on BAs, potentially because the focus is still on the generation of useful BA cell models rather than their further development. Using hanging drop spheroid formation followed by culture in ultra-low attachment plates, Klingelhutz et al. (76) demonstrated improved differentiation of murine BAT SVF compared with 2D. Alginate microstrands have also been used for the differentiation of BAs from murine embryonic stem cells and brown preadipocytes (77). Using 3D printed hyaluronic acid/gelatin gels, Kuss et al. (78) showed that whereas immortalized human WA progenitors prefer soft gels, immortalized human BA progenitors prefer stiffer gels, suggesting that developments within WA 3D culture methods cannot necessarily be directly translated to BA cultures.

### ECM and Vascularization

The next step toward recapitulating BAT *in vitro* is the addition of ECM and supporting cell types. It is possible to generate 3D scaffolds from ECM proteins such as collagens and glycoproteins (71), or to incorporate ECM components into synthetic hydrogels (72, 79). However, to recreate the human BAT ECM *in vitro*, its composition must first be fully characterized *in vivo*, in both physiological and pathophysiological states.

For the generation of functional models of AT, vascularization is an important parameter, which can also aid engraftment *in vivo* during transplantation studies, as shown (74). Angiogenic signals, including VEGF (vascular endothelial growth factor), play important roles not only in BAT function but also in the development of BAs (80–83). For instance, VEGFA is able to trigger local angiogenesis in BAT, leading to brown adipogenesis and upregulation of *Ucp1* and *Pgc1*α (81). Vascularized (white) adipocyte spheroids can be generated using AT SVF, which contains endothelial cells as well as preadipocytes, or by coculturing adipocytes with endothelial cells (74, 75). For the generation of vascularized human BA models, it may be advantageous to focus on coculture methods, to avoid the need for primary tissue. Similar challenges exist regarding the integration of immune cells into the BA cultures.

## Discussion

Our understanding of BAT in mice and humans has increased significantly over the last decade, and with this understanding, we are moving closer to the development of therapeutics targeting BAT for obesity treatment. One of the main limitations in the field is the limited understanding of human BAT, including its development and molecular signature. These questions are steadily being tackled through in-depth characterization of primary human BAT and the development of human *in vitro* BA models. Human BA models can be generated from immortalization of primary human BAs, from multipotent stem cells or hPSCs, among others, and each of these methods has distinct advantages and limitations. For instance, *in vitro* models generated from adult BAT may represent the most mature BA models, whereas hPSC-derived BAs may be less mature unless forward programming is employed. In contrast, hPSCs are useful for disease modeling involving genetic engineering or patient-specific genetic backgrounds. Without a defined molecular signature for human BAT, lacking due to the heterogeneity and disperse anatomical distribution of BAT depots, it may, however, be challenging to determine which *in vitro* model is the best. Furthermore, as BAT does not exclusively consist of BAs, true recapitulation of this tissue involves modeling of the BAT microenvironment, including physiological BA organization in 3D, crosstalk with supporting cells such as immune cells, vascularization, and an ECM to facilitate BA differentiation and cell–cell signaling.

The road toward the development of obesity therapeutics targeting human BAT thus has plenty of challenges. However, with continued development of BA models and characterization of the *in vivo* depots, these challenges may soon be overcome.

## Author Contributions

IS and AV-P wrote the manuscript. All authors contributed to the article and approved the submitted version.

## Conflict of Interest

The authors declare that the research was conducted in the absence of any commercial or financial relationships that could be construed as a potential conflict of interest.

## References

[1] World Health Organization Obesity and Overweight. (2020). Available online at: https://www.who.int/news-room/fact-sheets/detail/obesity-and-overweight (accessed March 18, 2020).

[2] Eckel RH Grundy SM Zimmet PZ. The metabolic syndrome. Lancet. (2005) 365:1415–28. 10.1016/S0140-6736(05)66378-7 15836891

[3] Kolb R Sutterwala FS Zhang W. Obesity and cancer: inflammation bridges the two. Curr Opin Pharmacol. (2016) 29:77–89. 10.1016/j.coph.2016.07.005 27429211 PMC4992602

[4] King LK March L Anandacoomarasamy A. Obesity and osteoarthritis. Indian J Med Res. (2013) 138:185–93. 24056594 PMC3788203

[5] Cannon B Nedergaard J. Brown adipose tissue: function and physiological significance. Physiol Rev. (2004) 84:277–359. 10.1152/physrev.00015.2003 14715917

[6] Bartelt A Bruns OT Reimer R Hohenberg H Ittrich H Peldschus K. Brown adipose tissue activity controls triglyceride clearance. Nat Med. (2011) 17:200–5. 10.1038/nm.2297 21258337

[7] Berbée JFP Boon MR Khedoe PPSJ Bartelt A Schlein C Worthmann A. Brown fat activation reduces hypercholesterolaemia and protects from atherosclerosis development. Nat Commun. (2015) 6:6356. 10.1038/ncomms7356 25754609 PMC4366535

[8] Liu X Zheng Z Zhu X Meng M Li L Shen Y. Brown adipose tissue transplantation improves whole-body energy metabolism. Cell Res. (2013) 23:851–4. 10.1038/cr.2013.64 23649313 PMC3674396

[9] Stanford KI Middelbeek RJW Townsend KL An D Nygaard EB Hitchcox KM. Brown adipose tissue regulates glucose homeostasis and insulin sensitivity. J Clin Invest. (2013) 123:215–23. 10.1172/JCI62308 23221344 PMC3533266

[10] Dewal RS Stanford KI. Effects of exercise on brown and beige adipocytes. Biochim Biophys Acta Mol Cell Biol Lipids. (2019) 1864:71–8. 10.1016/j.bbalip.2018.04.013 29684558 PMC6292667

[11] Cohen P Levy JD Zhang Y Frontini A Kolodin DP Svensson KJ. Ablation of PRDM16 and beige adipose causes metabolic dysfunction and a subcutaneous to visceral fat switch. Cell. (2014) 156:304–16. 10.1016/j.cell.2013.12.021 24439384 PMC3922400

[12] Seale P Conroe HM Estall J Kajimura S Frontini A Ishibashi J. Prdm16 determines the thermogenic program of subcutaneous white adipose tissue in mice. J Clin Invest. (2011) 121:96–105. 10.1172/JCI44271 21123942 PMC3007155

[13] Wu J Boström P Sparks LM Ye L Choi JH Giang A-H. Beige adipocytes are a distinct type of thermogenic fat cell in mouse and human. Cell. (2012) 150:366–76. 10.1016/j.cell.2012.05.016 22796012 PMC3402601

[14] Seale P Bjork B Yang W Kajimura S Chin S Kuang S. PRDM16 controls a brown fat/skeletal muscle switch. Nature. (2008) 454:961–7. 10.1038/nature07182 18719582 PMC2583329

[15] Cypess AM Lehman S Williams G Tal I Rodman D Goldfine AB. Identification and importance of brown adipose tissue in adult humans. N Engl J Med. (2009) 360:1509-17. 10.1056/NEJMoa0810780 19357406 PMC2859951

[16] Chondronikola M Volpi E Børsheim E Porter C Annamalai P Enerbäck S. Brown adipose tissue improves whole-body glucose homeostasis and insulin sensitivity in humans. Diabetes. (2014) 63:4089–99. 10.2337/db14-0746 25056438 PMC4238005

[17] Yoneshiro T Aita S Matsushita M Kayahara T Kameya T Kawai Y. Recruited brown adipose tissue as an antiobesity agent in humans. J Clin Invest. (2013) 123:3404–8. 10.1172/JCI67803 23867622 PMC3726164

[18] Leitner BP Huang S Brychta RJ Duckworth CJ Baskin AS McGehee S. Mapping of human brown adipose tissue in lean and obese young men. Proc Natl Acad Sci USA. (2017) 114:8649–54. 10.1073/pnas.1705287114 28739898 PMC5559032

[19] Kern PA Finlin BS Zhu B Rasouli N McGehee RE Westgate PM. The effects of temperature and seasons on subcutaneous white adipose tissue in humans: evidence for thermogenic gene induction. J Clin Endocrinol Metab. (2014) 99:E2772–9. 10.1210/jc.2014-2440 25299843 PMC4255113

[20] Kulterer OC Niederstaetter L Herz CT Haug AR Bileck A Pils D. The presence of active brown adipose tissue determines cold-induced energy expenditure and oxylipin profiles in humans. J Clin Endocrinol Metab. (2020) 105:2203–16. 10.1210/clinem/dgaa183 32343312

[21] Cypess AM White AP Vernochet C Schulz TJ Xue R Sass CA. Anatomical localization, gene expression profiling and functional characterization of adult human neck brown fat. Nat Med. (2013) 19:635–9. 10.1038/nm.3112 23603815 PMC3650129

[22] Sharp LZ Shinoda K Ohno H Scheel DW Tomoda E Ruiz L. Human BAT possesses molecular signatures that resemble beige/brite cells. PLoS ONE. (2012) 7:e49452. 10.1371/journal.pone.0049452 23166672 PMC3500293

[23] de Jong JMA Sun W Pires ND Frontini A Balaz M Jespersen NZ Human brown adipose tissue is phenocopied by classical brown adipose tissue in physiologically humanized mice. Nat Metab. (2019) 1:830–43. 10.1038/s42255-019-0101-4 32694768

[24] Kajimura S Spiegelman BM. Confounding issues in the ‘humanized’ BAT of mice. Nat Metab. (2020) 2:303–4. 10.1038/s42255-020-0192-y 32405619 PMC7220110

[25] Nedergaard J Cannon B. UCP1 mRNA does not produce heat. Biochim Biophys Acta. (2013) 1831:943–9. 10.1016/j.bbalip.2013.01.009 23353596

[26] Whittle AJ López M Vidal-Puig A Jia H Lubetkin EI Allison DB. Using brown adipose tissue to treat obesity - the central issue. Trends Mol Med. (2011) 17:405–11. 10.1016/j.molmed.2011.04.001 21602104

[27] Cypess AM Weiner LS Roberts-Toler C Franquet Elía E Kessler SH Kahn PA. Activation of human brown adipose tissue by a β3-adrenergic receptor agonist. Cell Metab. (2015) 21:33–8. 10.1016/j.cmet.2014.12.009 25565203 PMC4298351

[28] Riis-Vestergaard MJ Richelsen B Bruun JM Li W Hansen JB Pedersen SB. Beta-1 and not beta-3 adrenergic receptors may be the primary regulator of human brown adipocyte metabolism. J Clin Endocrinol Metab. (2020) 105:e994–1005. 10.1210/clinem/dgz298 31867674

[29] Hanssen MJW van der Lans AAJJ Brans B Hoeks J Jardon KMC Schaart G. Short-term cold acclimation recruits brown adipose tissue in obese humans. Diabetes. (2016) 65:1179–89. 10.2337/db15-1372 26718499

[30] Carobbio S Guénantin A-C Samuelson I Bahri M Vidal-Puig A. Brown and beige fat: from molecules to physiology and pathophysiology. Biochim Biophys Acta Mol Cell Biol Lipids. (2019) 1864:37–50. 10.1016/j.bbalip.2018.05.013 29852279

[31] Elsen M Raschke S Tennagels N Schwahn U Jelenik T Roden M. BMP4 and BMP7 induce the white-to-brown transition of primary human adipose stem cells. Am J Physiol Physiol. (2014) 306:C431–40. 10.1152/ajpcell.00290.2013 24284793

[32] Qian S-W Tang Y Li X Liu Y Zhang Y-Y Huang H-Y. BMP4-mediated brown fat-like changes in white adipose tissue alter glucose and energy homeostasis. Proc Natl Acad Sci USA. (2013) 110:E798–807. 10.1073/pnas.1215236110 23388637 PMC3587258

[33] Tseng Y Kokkotou E Schulz T Huang T Winnay J Taniguchi C. New role of bone morphogenetic protein 7 in brown adipogenesis and energy expenditure. Nature. (2008) 454:1000–4. 10.1038/nature07221 18719589 PMC2745972

[34] Whittle AJ Carobbio S Martins L Slawik M Hondares E Vázquez MJ. BMP8B increases brown adipose tissue thermogenesis through both central and peripheral actions. Cell. (2012) 149:871–85. 10.1016/j.cell.2012.02.066 22579288 PMC3383997

[35] Pellegrinelli V Peirce VJ Howard L Virtue S Türei D Senzacqua M. Adipocyte-secreted BMP8b mediates adrenergic-induced remodeling of the neuro-vascular network in adipose tissue. Nat Commun. (2018) 9:1–18. 10.1038/s41467-018-07453-x 30478315 PMC6255810

[36] Fisher FM Kleiner S Douris N Fox EC Mepani RJ Verdeguer F. FGF21 regulates PGC-1α and browning of white adipose tissues in adaptive thermogenesis. Genes Dev. (2012) 26:271–81. 10.1101/gad.177857.111 22302939 PMC3278894

[37] Chen MZ Chang JC Zavala-Solorio J Kates L Thai M Ogasawara A. FGF21 mimetic antibody stimulates UCP1-independent brown fat thermogenesis via FGFR1/βKlotho complex in non-adipocytes. Mol Metab. (2017) 6:1454–67. 10.1016/j.molmet.2017.09.003 29107292 PMC5681280

[38] Zhang Y Xie C Wang H Foss RM Clare M George EV. Irisin exerts dual effects on browning and adipogenesis of human white adipocytes. Am J Physiol Endocrinol Metab. (2016) 311:E530–41. 10.1152/ajpendo.00094.2016 27436609

[39] Elsen M Raschke S Eckel J. Browning of white fat: does irisin play a role in humans? J Endocrinol. (2014) 222:R25–38. 10.1530/JOE-14-0189 24781257

[40] Arhire LI Mihalache L Covasa M. Irisin: a hope in understanding and managing obesity and metabolic syndrome. Front Endocrinol. (2019) 10:524. 10.3389/fendo.2019.00524 31428053 PMC6687775

[41] Seiler SE Xu D Ho J-P Lo KA Buehrer BM Ludlow YJW. Characterization of a primary brown adipocyte culture system derived from human fetal interscapular fat. Adipocyte. (2015) 4:303–10. 10.1080/21623945.2015.1042192 26451287 PMC4573190

[42] Zilberfarb V Piétri-Rouxel F Jockers R Krief S Delouis C Issad T. Human immortalized brown adipocytes express functional beta3-adrenoceptor coupled to lipolysis. J Cell Sci. (1997) 110:801–7. 9133667 10.1242/jcs.110.7.801

[43] Shinoda K Luijten IHN Hasegawa Y Hong H Sonne SB Kim M. Genetic and functional characterization of clonally derived adult human brown adipocytes. Nat Med. (2015) 21:389–94. 10.1038/nm.3819 25774848 PMC4427356

[44] Xue R Lynes MD Dreyfuss JM Shamsi F Schulz TJ Zhang H. Clonal analyses and gene profiling identify genetic biomarkers of the thermogenic potential of human brown and white preadipocytes. Nat Med. (2015) 21:760–8. 10.1038/nm.3881 26076036 PMC4496292

[45] Markussen LK Isidor MS Breining P Andersen ES Rasmussen NE Petersen LI. Characterization of immortalized human brown and white pre-adipocyte cell models from a single donor. PLoS ONE. (2017) 12:e0185624. 10.1371/journal.pone.0185624 28957413 PMC5619805

[46] Kishida T Ejima A Yamamoto K Tanaka S Yamamoto T Mazda O. Reprogrammed functional brown adipocytes ameliorate insulin resistance and dyslipidemia in diet-induced obesity and type 2 diabetes. Stem Cell Rep. (2015) 5:569. 10.1016/j.stemcr.2015.08.007 26365511 PMC4624936

[47] Takeda Y Harada Y Yoshikawa T Dai P. Direct conversion of human fibroblasts to brown adipocytes by small chemical compounds. Sci Rep. (2017) 7:4304. 10.1038/s41598-017-04665-x 28655922 PMC5487346

[48] Nedergaard J Petrovic N Lindgren EM Jacobsson A Cannon B. PPARγ in the control of brown adipocyte differentiation. Biochim Biophys Acta Mol Basis Dis. (2005) 1740:293–304. 10.1016/j.bbadis.2005.02.003 15949696

[49] Crisan M Casteilla L Lehr L Carmona M Paoloni-Giacobino A Yap S. A reservoir of brown adipocyte progenitors in human skeletal muscle. Stem Cells. (2008) 26:2425–33. 10.1634/stemcells.2008-0325 18617684

[50] Ullah I Subbarao RB Rho GJ. Human mesenchymal stem cells - current trends and future prospective. Biosci Rep. (2015) 35:e00191. 10.1042/BSR20150025 25797907 PMC4413017

[51] Elabd C Chiellini C Carmona M Galitzky J Cochet O Petersen R. Human multipotent adipose-derived stem cells differentiate into functional brown adipocytes. Stem Cells. (2009) 27:2753–60. 10.1002/stem.200 19697348

[52] Rashnonejad A Ercan G Gunduz C Akdemir A Tiftikcioglu YO. Comparative analysis of human UCB and adipose tissue derived mesenchymal stem cells for their differentiation potential into brown and white adipocytes. Mol Biol Rep. (2018) 45:233–44. 10.1007/s11033-018-4156-1 29453764

[53] Huang P-I Chen Y-C Chen L-H Juan C-C Ku H-H Wang S-T. PGC-1α mediates differentiation of mesenchymal stem cells to brown adipose cells. J Atheroscler Thromb. (2011) 18:966–80. 10.5551/jat.7401 21817823

[54] Clevers H. Modeling development and disease with organoids. Cell. (2016) 165:1586–97. 10.1016/j.cell.2016.05.082 27315476

[55] Takahashi K Tanabe K Ohnuki M Narita M Ichisaka T Tomoda K. Induction of pluripotent stem cells from adult human fibroblasts by defined factors. Cell. (2007) 131:861–72. 10.1016/j.cell.2007.11.019 18035408

[56] Ahfeldt T Schinzel RT Lee Y-K Hendrickson D Kaplan A Lum DH. Programming human pluripotent stem cells into white and brown adipocytes. Nat Cell Biol. (2012) 14:209–19. 10.1038/ncb2411 22246346 PMC3385947

[57] Lamarche É Lala-Tabbert N Gunanayagam A St-Louis C Wiper-Bergeron N. Retinoic acid promotes myogenesis in myoblasts by antagonizing transforming growth factor-beta signaling via C/EBPβ. Skelet Muscle. (2015) 5:8. 10.1186/s13395-015-0032-z 25878769 PMC4397812

[58] Pawlowski M Ortmann D Bertero A Tavares JM Pedersen RA Vallier L. Inducible and deterministic forward programming of human pluripotent stem cells into neurons, skeletal myocytes, and oligodendrocytes. Stem Cell Rep. (2017) 8:803. 10.1016/j.stemcr.2017.02.016 28344001 PMC5390118

[59] Moreau T Evans AL Vasquez L Tijssen MR Yan Y Trotter MW. Large-scale production of megakaryocytes from human pluripotent stem cells by chemically defined forward programming. Nat Commun. (2016) 7:11208. 10.1038/ncomms11208 27052461 PMC4829662

[60] Zhang Y Pak C Han Y Ahlenius H Zhang Z Chanda S. Rapid single-step induction of functional neurons from human pluripotent stem cells. Neuron. (2013) 78:785–98. 10.1016/j.neuron.2013.05.029 23764284 PMC3751803

[61] Mohsen-Kanson T Hafner A-L Wdziekonski B Takashima Y Villageois P Carrière A. Differentiation of human induced pluripotent stem cells into brown and white adipocytes: role of pax3. Stem Cells. (2014) 32:1459–67. 10.1002/stem.1607 24302443

[62] Hafner A-L Contet J Ravaud C Yao X Villageois P Suknuntha K. Brown-like adipose progenitors derived from human induced pluripotent stem cells: identification of critical pathways governing their adipogenic capacity. Sci Rep. (2016) 6:32490. 10.1038/srep32490 27577850 PMC5006163

[63] van Meeteren LA ten Dijke P. Regulation of endothelial cell plasticity by TGF-β. Cell Tissue Res. (2012) 347:177–86. 10.1007/s00441-011-1222-6 21866313 PMC3250609

[64] Nishio M Yoneshiro T Nakahara M Suzuki S Saeki K Hasegawa M. Production of functional classical brown adipocytes from human pluripotent stem cells using specific hemopoietin cocktail without gene transfer. Cell Metab. (2012) 16:394–406. 10.1016/j.cmet.2012.08.001 22958922

[65] Oka M Kobayashi N Matsumura K Nishio M Saeki K. Exogenous cytokine-free differentiation of human pluripotent stem cells into classical brown adipocytes. Cells. (2019) 8:373. 10.3390/cells8040373 31022954 PMC6523334

[66] Pope BD Warren CR Parker KK Cowan CA. Microenvironmental control of adipocyte fate and function. Trends Cell Biol. (2016) 26:745–55. 10.1016/j.tcb.2016.05.005 27268909 PMC6788628

[67] Sun K Tordjman J Clément K Scherer PE. Fibrosis and adipose tissue dysfunction. Cell Metab. (2013) 18:470–7. 10.1016/j.cmet.2013.06.016 23954640 PMC3795900

[68] Shoham N Gefen A. Mechanotransduction in adipocytes. J Biomech. (2012) 45:1–8. 10.1016/j.jbiomech.2011.10.023 22112919

[69] Chun T-H Hotary KB Sabeh F Saltiel AR Allen ED Weiss SJ. A pericellular collagenase directs the 3-dimensional development of white adipose tissue. Cell. (2006) 125:577–91. 10.1016/j.cell.2006.02.050 16678100

[70] Unser AM Tian Y Xie Y. Opportunities and challenges in three-dimensional brown adipogenesis of stem cells. Biotechnol Adv. (2015) 33:962–79. 10.1016/j.biotechadv.2015.07.005 26231586 PMC4562467

[71] Murphy CS Liaw L Reagan MR. In vitro tissue-engineered adipose constructs for modeling disease. BMC Biomed Eng. (2019) 1:27. 10.1186/s42490-019-0027-7 32133436 PMC7055683

[72] Pellegrinelli V Heuvingh J du Roure O Rouault C Devulder A Klein C. Human adipocyte function is impacted by mechanical cues. J Pathol. (2014) 233:183–95. 10.1002/path.4347 24623048

[73] Harms MJ Li Q Lee S Zhang C Kull B Hallen S. Mature human white adipocytes cultured under membranes maintain identity, function, and can transdifferentiate into brown-like adipocytes. Cell Rep. (2019) 27:213–25.e5. 10.1016/j.celrep.2019.03.026 30943403

[74] Muller S Ader I Justine C Leménager H Achard P Casteilla L. Human adipose stromal-vascular fraction self-organizes to form vascularized adipose tissue in 3D cultures. (2019) 9:7520. 10.1038/s41598-019-43624-6 31076601 PMC6510792

[75] Daquinag AC Souza GR Kolonin MG. Adipose tissue engineering in three-dimensional levitation tissue culture system based on magnetic nanoparticles. Tissue Eng Part C Methods. (2013) 19:336–44. 10.1089/ten.tec.2012.0198 23017116 PMC3603558

[76] Klingelhutz AJ Gourronc FA Chaly A Wadkins DA Burand AJ Markan KR. Scaffold-free generation of uniform adipose spheroids for metabolism research and drug discovery. Sci Rep. (2018) 8:523. 10.1038/s41598-017-19024-z 29323267 PMC5765134

[77] Unser AM Mooney B Corr DT Tseng YH Xie Y. 3D brown adipogenesis to create “Brown-Fat-in-Microstrands.” *Biomaterials*. (2016). 75:123–34. 10.1016/j.biomaterials.2015.10.017 26496384 PMC4644497

[78] Kuss M Kim J Qi D Wu S Lei Y Chung S. Effects of tunable, 3D-bioprinted hydrogels on human brown adipocyte behavior and metabolic function. Acta Biomater. (2018) 71:486–95. 10.1016/j.actbio.2018.03.021 29555462 PMC6066177

[79] Louis F Pannetier P Souguir Z Le Cerf D Valet P Vannier J-P. A biomimetic hydrogel functionalized with adipose ECM components as a microenvironment for the 3D culture of human and murine adipocytes. Biotechnol Bioeng. (2017) 114:1813–24. 10.1002/bit.26306 28398656

[80] Bagchi M Kim LA Boucher J Walshe TE Kahn CR D'Amore PA. Vascular endothelial growth factor is important for brown adipose tissue development and maintenance. FASEB J. (2013) 27:3257–71. 10.1096/fj.12-221812 23682123 PMC3714576

[81] Sun K Kusminski CM Luby-Phelps K Spurgin SB An YA Wang QA. Brown adipose tissue derived VEGF-A modulates cold tolerance and energy expenditure. Mol Metab. (2014) 3:474–83. 10.1016/j.molmet.2014.03.010 24944907 PMC4060212

[82] Shimizu I Aprahamian T Kikuchi R Shimizu A Papanicolaou KN MacLauchlan S. Vascular rarefaction mediates whitening of brown fat in obesity. J Clin Invest. (2014) 124:2099–112. 10.1172/JCI71643 24713652 PMC4001539

[83] Min SY Kady J Nam M Rojas-Rodriguez R Berkenwald A Kim JH. Human “brite/beige” adipocytes develop from capillary networks, and their implantation improves metabolic homeostasis in mice. Nat Med. (2016) 22:312–8. 10.1038/nm.4031 26808348 PMC4777633

